# Comparison of score-based prediction of 90-day mortality after liver resection

**DOI:** 10.1186/s12893-020-0678-2

**Published:** 2020-01-29

**Authors:** Tanja Knoblich, Ulf Hinz, Christos Stravodimos, Michael R. Schön, Arianeb Mehrabi, Markus W. Büchler, Katrin Hoffmann

**Affiliations:** 1 Department of General, Visceral and Transplant Surgery, Ruprecht-Karls University, Im Neuenheimer Feld 110, 69120 Heidelberg, Germany; 2 Department of General and Visceral Surgery, Städtisches Klinikum, Moltkestraße 90, 76133 Karlsruhe, Germany

**Keywords:** Liver resection, Mortality, Prognostic score

## Abstract

**Background:**

Indications for liver surgery are expanding fast and complexity of procedures increases. Preoperative mortality risk assessment by scoring systems is debatable. A previously published externally validated Mortality Risk Score allowed easy applicable and precise prediction of postoperative mortality. Aim of the study was to compare the performance of the Mortality Risk Score with the standard scores MELD and P-POSSUM.

**Methods:**

Data of 529 patients undergoing liver resection were analysed. Mortality Risk Score, the labMELD Score and the P-POSSUM Scores (PS, OS, P-POSSUM mortality %) were calculated. The ROC curves of the three scoring systems were computed and the areas under the curve (C-index) were calculated using logistic regression models. Comparisons between the ROC curves were performed using the corresponding Wald tests.

**Results:**

Internal validation confirmed that the risk model was predictive for a 90-day mortality rate with a C-index of 0.8421. The labMELD Score had a C-index of 0.7352 and the P-POSSUM system 0.6795 (PS 0.6953, OS 0.5413). The 90-day mortality rate increased with increasing labMELD values (*p* < 0.0001). Categorized according to the Mortality Risk Score Groups the labMELD Score showed a linear increase while the POSSUM Scores showed variable results.

**Conclusions:**

By accurately predicting the risk of postoperative mortality after liver surgery the Mortality Risk Score should be useful at the selection stage. Prediction can be adjusted by use of the well-established labMELD Score. In contrast, the performance of standard P-POSSUM Scores is limited.

## Background

Indications for liver surgery are expanding fast and the complexity of procedures has generally increased over the years. The preoperative assessment of the safety of liver surgery and identification of high-risk patients remains challenging. Multiple patient-related factors and procedure-specific variables directly influence the perioperative outcome as well as the 90-day mortality after hepatectomy.

To provide appropriate counseling to the patient regarding the risks and outcome of the planned procedure is the crucial point within a shared decision-making process. Quality of life after surgery has increasingly become an important patient-related endpoint after surgical interventions. Nevertheless, the surgeons’ intuition is not the best when it comes to predicting the outcome after liver resection and “experience-based” indications are no longer acceptable [1, 2].

Various scoring systems such as MELD Score, E-PASS, mE-PASS, Portsmouth-Physiological and Operative Severity Score for the enUmeration of Mortality and morbidity (P-POSSUM), Child-Turcotte-Pugh score or ASA classification have been used in an attempt to accurately predict mortality in liver surgery. However, the results were disappointing. General surgical risk models (E-PASS, mE-PASS and P-POSSUM) tended to overestimate the mortality rate by more than twofold [3]. For prognostication of patients with known liver disease the utility of MELD Score has been well documented. However, heterogenic reports are available regarding its ability to predict morbidity or mortality after elective liver surgery [4, 5]. The P-POSSUM Score is now the most widely validated predictive scoring system used in perioperative care. P-POSSUM aims to calculate the risk of a surgical procedure based on the patient’s physiological condition and operative parameters. In hepato-biliary-pancreatic surgery the predictive value of P-POSSUM is under debate [3, 6, 7]. Previously, we suggested an easy applicable externally validated scoring system for 90-day mortality [8]. The aim of the current study is to compare its performance with already established scoring systems.

## Methods

Data of 529 patients undergoing liver resection between 2014 and 2016 at the Department of General and Transplant Surgery, University Hospital Heidelberg were analysed. The institution’s liver resection database contained anonymous information for about 3447 cases performed between 2001 and 2018, which represented more than 99% of all liver resections performed in the department. Patients who underwent liver resection for hepatobiliary trauma as well as after liver transplantation and patients who underwent resections in conjunction with other operations (such as pancreaticoduodenectomy, laparoscopic resections or the deroofing of simple or parasitic cysts, cystectomy and necrosectomy) were excluded from the analysis. Resections performed in patients younger than 18 years of age were also excluded. Written informed consent was obtained by every patient included in the analysis and the study was approved by the ethical committee of the Medical Faculty of Ruprecht-Karls-University Heidelberg S-557/2017.

For all patients the previously suggested Mortality Risk Score, the labMELD Score and the P-POSSUM Score (PS, OS, P-POSSUM mortality %) were calculated.

As described previously the Mortality Risk Score was composed of 8 different parameters. One point was assigned for each of the following factors: patient’s age ≥ 60 years, the performance of right trisectionectomy, preoperative INR ≥ 1.1 and preoperative GGT values ≥60 U/l. Absence of these factors resulted in an assignment of 0 points. Two points were assigned for a preoperative platelet count value < 120/nl (preoperative platelet count values ≥120/nl, 0 points). Three points were assigned for a preoperative creatinine value ≥2 mg/dl (preoperative creatinine values < 2 mg/dl, 0 points). The histological diagnosis was stratified according to a 2-point scale; intrahepatic cholangiocarcinoma (1 point), perihilar cholangiocarcinoma (2 points) and all other diagnoses (0 points). ASA classification was stratified according to: ASA IV (5 points), ASA III (1 point) and ASA I or II (0 points). Based on this the patients could be divided into four different Risk Score Groups. Risk Score Group 1 (very low-risk, 0–1 points), Risk Score Group 2 (low-risk, 2–3 points), Risk Score Group 3 (medium-risk, 4–5 points), and Risk Score Group 4 (high-risk, ≥ 6 points) [8]. The labMELD Score was calculated using serum bilirubin, serum creatinine and International Normalized Ratio (INR) according to the following formula: LabMELD = 10x (0.957 x ln (serum creatinine mg/dl) + 0.378 x ln (serum bilirubin mg/dl) + 1.120 x ln (INR) + 0.643). Serum creatinine was capped at 4 mg/dl. For patients receiving dialysis at least 2 times in the previous week the creatinine value was set to 4 mg/dl. The value was rounded to the nearest integer. In order to avoid negative scores, the lower limit for each component of the score was set to 1. LabMELD Score values between 6 and 40 were possible [9–12]. The P-POSSUM equation for in-patient mortality was according to Prytherch et al.: ln (R/(1-R)) = − 9.065 + (0.1692 x physiological score) + (0.1550 x operative severity score). R is the predicted risk of mortality [13]. The score points of the physiological score and the operative severity score were awarded on the basis of the original research study of Copeland et al. [14] For the study population the 12 physiological parameters (age, cardiac history, respiratory history, systolic blood pressure, pulse rate, Glasgow Coma Scale, hemoglobin level, white cell count, urea concentration, sodium level, potassium level and electrocardiography) and the six operative parameters (operative severity, multiple procedures, total blood loss, peritoneal soiling, presence of malignancy and mode of surgery) were analysed and the physiological score (PS), operative severity score (OS) and P-POSSUM mortality % were calculated. For all patients the operative severity was set to major.

### Statistical analysis

SAS software (Release 9.4, SAS Institute, Cary, NC, USA) was used for statistical data analysis. Categorical parameters were presented as absolute and relative frequencies. Differences between the 90-day mortality subgroup and the rest of the total cohort were analysed using Fisher’s exact test. Continuous parameters were expressed as the median, the interquartile range (IQR), the mean and the standard deviation (SD). With respect to the continuous parameters, comparisons between the 90-day mortality subgroup and the rest of the total cohort were performed using the Mann-Whitney U test. Logistic regression analyses were performed in order to examine the categorized Risk Score and the categorized MELD Score with respect to the 90-day mortality. Odds ratios (OR) with their corresponding 95% confidence intervals (CI) are presented here. The receiver operating characteristic (ROC) curves of the three scoring systems Mortality Risk Score, MELD Score, and P-POSSUM Score were computed and the areas under the curve (known as the C-index) were calculated using logistic regression models. Comparisons between the ROC curves were performed using the corresponding Wald tests from the underlying logistic regression models. Two-sided *p*-values of less than 0.05 were considered statistically significant.

## Results

### Patients’ characteristics and risk profile

Data of 529 patients undergoing liver resection between 2014 and 2016 at the Department of General and Transplant Surgery, University Hospital Heidelberg were analysed for internal validation of the previously reported risk score [8]. The median age was 61.8 years (IQR from 52.8–69.0) and 61.1% (*n* = 323) of cases were male and 38.9% (*n* = 206) female. American Society of Anesthesiologists (ASA) classification of ≥ III was found in 45.9% (*n* = 243) of cases. A BMI ≥ 30 kg/m^2^ was evident in 18.8% (*n* = 98). Major co-morbidities were: diabetes 17.6% (*n* = 93), cardiac disease 19.3% (*n* = 102), pulmonary disease 8.9% (*n* = 47) and renal disease 6.2% (*n* = 33). The further relevant factors for the risk model were the performance of right trisectionectomy (*n* = 39, 7.4%), intrahepatic cholangiocarcinoma (*n* = 59, 11.2%), perihilar cholangiocarcinoma (*n* = 35, 6.6%), preoperative INR ≥ 1.1 (*n* = 61, 11.5%), preoperative GGT ≥ 60 U/l (*n* = 285, 53.9%), preoperative platelet count value < 120/nl (*n* = 32, 6.0%), preoperative creatinine value ≥2 mg/dl (*n* = 7, 1.3%) and a total bilirubin ≥2 mg/dl (*n* = 33, 6.2%). Details of the study population and the *p*-values are shown in Table 1.

**Table 1:** Score relevant patients’ characteristics

Characteristics	Total Cohort (*n* = 529)		90-day Mortality (*n* = 24, 4.54%)		*P*-Value
Demographics	*n*	%	*n*	%	
Age (median; IQR)	61.8	(52.8–69.0)	69.3	(62.1–74.9)	0.0049
Male: Female	323:206	61.1%:38.9%	20:4	83.3%:16.7%	0.0301
Preoperative risk					
Diabetes mellitus	93	17.6%	8	33.3%	0.0516
Cardiac disease	102	19.3%	8	33.3%	0.1064
Renal disease	33	6.2%	2	8.3%	0.6557
Pulmonary disease	47	8.9%	1	4.2%	0.7125
ASA III,IV	243	45.9%	14	58.3%	0.2945
BMI ≥ 30 kg/m^2a^	98	18.8%	3	13.0%	0.5939
Tumour entity					
Intrahepatic cholangiocarcinoma	59	11.2%	2	8.3%	1.0
Perihilar cholangiocarcinoma	35	6.6%	8	33.3%	< 0.0001
Surgical procedure					
Right trisectionectomy	39	7.4%	10	41.7%	< 0.0001
Preoperative lab values					
INR ≥ 1.1	61	11.5%	6	25.0%	0.0466
GGT ≥ 60 U/l	285	53.9%	22	91.7%	< 0.0001
Thrombocytes < 120/nl	32	6.0%	4	16.7%	0.0497
Creatinine≥2 mg/dl	7	1.3%	1	4.2%	0.2789
Total Bilirubin≥2 mg/dl	33	6.2%	7	29.2%	0.0003

^a^ missing values n = 7

### 90-day mortality rate and internal validation

The 90-day mortality rate of the study population was 4.5% (*n* = 24). Internal validation of the risk model confirmed that the risk score points were predictive for a 90-day mortality rate with a C-index of 0.8421. The mortality rate was 0.5% for patients having 0 to 1 point (*n* = 1, very low-risk group 1, OR 1), 0.8% for those having 2 points (n = 1, low-risk group 2A, OR 0.50, 95% CI 0.03–3.98, *p*-value 0.5547), 6.1% for those having 3 points (*n* = 6, low-risk group 2B, OR 4.19, 95% CI 1.08–20.21, *p*-value 0.0459), 9.1% for those having 4 points (*n* = 5, medium-risk group 3A, OR 6.50, 95% CI 1.54–32.55, *p*-value 0.0123), 9.4% for those having 5 points (*n* = 3, medium-risk group 3B, OR 6.72, 95% CI 1.20–37.86, p-value 0.0234) and 40.0% for those having ≥6 points (*n* = 8, high-risk group 4, OR 43.33, 95% CI 9.87–234.14, p-value < 0.0001). Table 2.

**Table 2:** 90-day mortality rate and internal validation of Heidelberg Mortality Risk Score

Mortality Risk Score Points	Risk Score Groups	N total	N 90-day mortality	% 90-day mortality	OR	95% CI	*p*-value
0 + 1	1 (very low-risk)	193	1	0.5	1		
2	2A (low-risk)	130	1	0.8	0.50	0.03–3.98	0.5547
3	2B (low-risk)	99	6	6.1	4.19	1.08–20.21	0.0459
4	3A (medium-risk)	55	5	9.1	6.50	1.54–32.55	0.0123
5	3B (medium-risk)	32	3	9.4	6.72	1.20–37.86	0.0234
≥6	4 (high-risk)	20	8	40.0	43.33	9.87–234.14	< 0.0001

*OR* Odds ratio *CI* Confidence interval

### Comparison with labMELD score and P-POSSUM scores

The median labMELD Score of the study population of the 529 patients was 7.0 (IQR 6–8) and the mean labMELD Score was 7.5 (SD 2.7). The 90-day mortality rates according to the labMELD Score were as following: labMELD Score 6 1.7% (*n* = 4 of 236, OR1), labMELD Score 7 3.4% (*n* = 5 of 148, OR 2.03, 95% CI 0.53–8.31, *p*-value 0.2979), labMELD Score 8 7.0% (*n* = 4 of 57, OR 4.38, 95% CI 1.01–19.05, *p*-value 0.0412), labMELD Score 9 and 10 4.9% (*n* = 2 of 41, OR 2.97, 95% CI 0.40–15.78, p-value 0.2171), labMELD Score 11–14 14.3% (n = 4 of 28, OR 9.67, 95% CI 2.17–43.31, p-value 0.0021) and labMELD Score ≥ 15 26.3% (*n* = 5 of 19, OR 20.71, 95% CI 4.98–92.32, p-value < 0.0001). Table 3. The labMELD Score was predictive for a 90-day mortality rate with a C-index of 0.7352.

**Table 3:** 90-day mortality rate according to labMELD Score

labMELD Score points	N total	N (%) 90-day mortality	OR	95% CI	*p*-value
6	236	4 (1.7)	1		
7	148	5 (3.4)	2.03	0.53–8.31	0.2979
8	57	4 (7.0)	4.38	1.01–19.05	0.0412
9 + 10	41	2 (4.9)	2.97	0.40–15.78	0.2171
11–14	28	4 (14.3)	9.67	2.17–43.31	0.0021
≥15	19	5 (26.3)	20.71	4.98–92.32	< 0.0001

*OR* Odds ratio *CI* Confidence interval

**Fig. 1:**
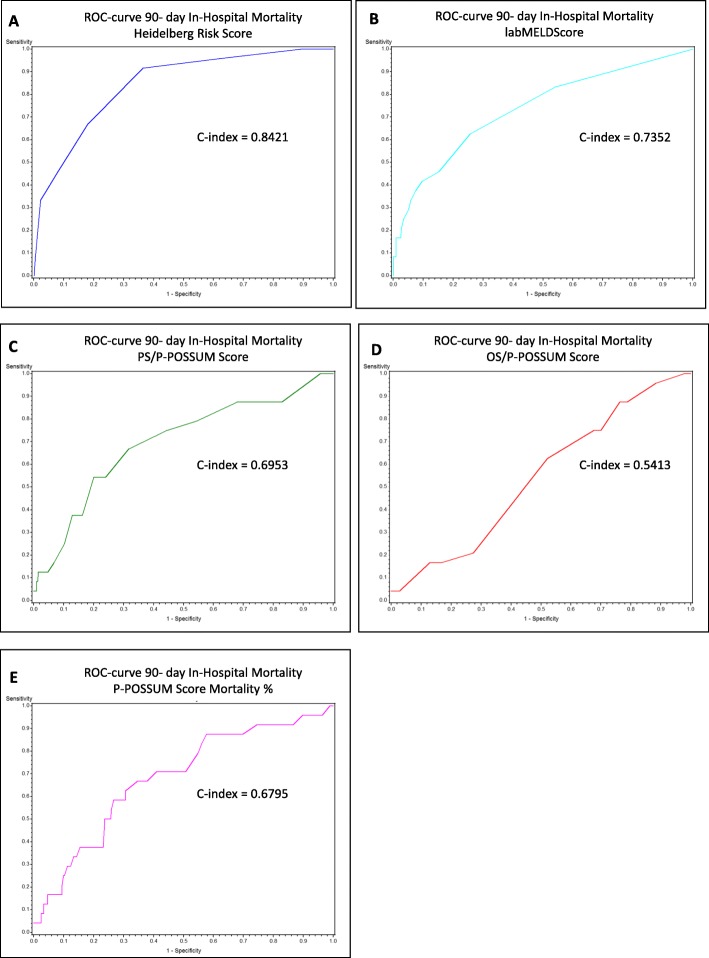
Receiver operating characteristic (ROC) curve obtained for (**a**) Mortality Risk Score; (**b**) labMELD Score; (**c**) PS/P-POSSUM Score; (**d**) OS/P-POSSUM Score and (**e**) P-POSSUM mortality % to predict the 90-day mortality rate in 529 patients after liver resection

The P-POSSUM scoring system consists of two parts, the physiological score (PS) and the operative severity score (OS). The mortality rate is defined as in-patient mortality. The median PS was 16.0 (IQR 14–18.8) and the mean PS was 17.1 (SD 4.5). The median OS was 17.0 (IQR 13–19) and the mean OS was 16.2 (SD 4.2). The median mortality rate of the P-POSSUM scoring system amounted to 2.3% (IQR 1.4–4.4) and the mean mortality rate amounted to 4.3% (SD 7.3). The C-value for AUC for the prediction of in-patient mortality for the PS part of P-POSSUM scoring system was 0.6953, for the OS part 0.5413 and for the mortality rate of the P-POSSUM system 0.6795 Table 4 Fig. 1.

**Table 4:** AUC value of the Mortality Risk Score compared to the AUC values of the labMELD Score and the P-POSSUM Scores

Risk score	AUC value	95% CI	*p*-value
Mortality Risk Score	0.8421	0.7675–0.9167	
labMELD Score	0.7352	0.6252–0.8452	0.0163
PS/P-POSSUM	0.6953	0.5794–0.8112	0.0278
OS/P-POSSUM	0.5413	0.4348–0.6478	< 0.0001
P-POSSUM mortality %	0.6795	0.5682–0.7908	0.0166

*AUC* Area under the curve *CI* Confidence interval

### Performance of labMELD score and P-POSSUM scores according to mortality risk score groups

The median labMELD Scores and mean labMELD Scores categorized according to Mortality Risk Score Groups were Risk Score Group 1 (very low-risk) *n* = 193 median labMELD 6.0 (IQR 6–7) and mean labMELD 6.5 (SD 0.8); Group 2A (low-risk) *n* = 130 median labMELD 7.0 (IQR 6–8) and mean labMELD 7.0 (SD 1.4); Group 2B (low-risk) *n* = 99 median labMELD 7.0 (IQR 6–8) and mean labMELD 7.7 (SD 2.4); Group 3A (medium-risk) *n* = 55 median labMELD 8.0 (IQR 7–9) and mean labMELD 8.4 (SD 2.8); Group 3B (medium-risk) *n* = 32 median labMELD 9.0 (IQR 7–13) and mean labMELD 10.6 (SD 4.0) and Group 4 (high-risk) *n* = 20 median labMELD 13.0 (IQR 8–17) and mean labMELD 13.4 (SD 6.1), *p*-value < 0.0001.

The median and mean PS categorized according to Mortality Risk Score Groups were Risk Score Group 1 (very low-risk) median PS 15.0 (IQR 13–17) and mean PS 15.4 (SD 2.8); Group 2A (low-risk) median PS 16.0 (IQR 14–18) and mean PS 16.8 (SD 3.7); Group 2B (low-risk) median PS 17.0 (IQR 15–21) and mean PS 19.1 (SD 6.2); Group 3A (medium-risk) median PS 17.0 (IQR 15–21) and mean PS 18.6 (SD 4.8); Group 3B (medium-risk) median PS 18.0 (IQR 16–23) and mean PS 19.2 (SD 5.2) and Group 4 (high-risk) median PS 19.0 (IQR 19–24) and mean PS 22.0 (SD 10.5), *p*-value < 0.0001. The median and mean OS categorized according to Mortality Risk Score Groups were Risk Score Group 1 (very low-risk) median OS 16.0 (IQR 12–19) and mean OS 16.0 (SD 4.1); Group 2A (low-risk) median OS 17.0 (IQR 15–19) and mean OS 16.7 (SD 3.9); Group 2B (low-risk) median OS 17.0 (IQR 13–19) and mean OS 16.8 (SD 4.5); Group 3A (medium-risk) median OS 16.0 (IQR 11–17) and mean OS 15.7 (SD 4.5); Group 3B (medium-risk) median OS 13.0 (IQR 11–17) and mean OS 14.2 (SD 4.2) and Group 4 (high-risk) median OS 17.0 (IQR 13–19) and mean OS 17.3 (SD 5.2), *p*-value 0.0132. The median and mean mortality rates of the P-POSSUM system were for Risk Score Group 1 (very low-risk) median mortality rate 1.7% (IQR 1.2–2.8) and mean mortality rate 2.9% (SD 4.5); for Risk Score Group 2A (low-risk) median mortality rate 2.4% (IQR 1.4–4.9) and mean mortality rate 4.0% (SD 5.3); for Risk Score Group 2B (low-risk) median mortality rate 3.3% (IQR 1.9–6.8) and mean mortality rate 6.6% (SD 11.3); for Risk Score Group 3A (medium-risk) median mortality rate 2.8% (IQR 1.1–5.6) and mean mortality rate 5.6% (SD 9.5); for Risk Score Group 3B (medium-risk) median mortality rate 1.7% (IQR 1.1–5.2) and mean mortality rate 5.0% (SD 7.6) and for Risk Score Group 4 (high-risk) median mortality rate 3.4% (IQR 2.4–5.3) and mean mortality rate 13.2% (SD 29.0), *p*-value < 0.0001. Table 5.

**Table 5:** Comparison of different scoring systems according to the 90-day Mortality Risk Score groups

Mortality Risk Score		labMELD Score		P-POSSUM % mortality		PS/P-POSSUM		OS/P-POSSUM	
Score Points	Score Group	Median Score (IQR)	Mean Score (SD)	Median Score (IQR)	Mean Score (SD)	Median Score (IQR)	Mean Score (SD)	Median Score (IQR)	Mean Score (SD)
0 + 1	1 (very low-risk)	6 (6–7)	6.5 (0.8)	1.7 (1.2–2.8)	2.9 (4.5)	15 (13–17)	15.4 (2.8)	16 (12–19)	16.0 (4.1)
2	2A (low-risk)	7 (6–8)	7.0 (1.4)	2.4 (1.4–4.9)	4.0 (5.3)	16 (14–18)	16.8 (3.7)	17 (15–19)	16.7 (3.9)
3	2B (low-risk)	7 (6–8)	7.7 (2.4)	3.3 (1.9–6.8)	6.6 (11.3)	17 (15–21)	19.1 (6.2)	17 (13–19)	16.8 (4.5)
4	3A (medium-risk)	8 (7–9)	8.4 (2.8)	2.8 (1.1–5.6)	5.6 (9.5)	17 (15–21)	18.6 (4.8)	16 (11–17)	15.7 (4.5)
5	3B (medium-risk)	9 (7–13)	10.6 (4.0)	1.7 (1.1–5.2)	5.0 (7.6)	18 (16–23)	19.2 (5.2)	13 (11–17)	14.2 (4.2)
≥6	4 (high-risk)	13 (8–17)	13.4 (6.1)	3.4 (2.4–5.3)	13.2 (29.0)	19 (19–24)	22.0 (10.5)	17 (13–19)	17.3 (5.2)
*p*-value		< 0.0001		< 0.0001		< 0.0001		0.0132	

## Discussions

Selection criteria for hepatectomy and prediction of the individual mortality risk are debatable. This study aimed at evaluating prognostic models of postoperative mortality in patients undergoing hepatectomy. By accurately predicting the risk of 90-day mortality the Mortality Risk Score should be useful at the selection stage. Using only preoperatively available parameters it is easy applicable and does not need subjective judgement of patients’ condition.

Several risk assessment scores have been proposed for the prediction of outcomes after liver resection previously [15–24]. The labMELD and P-POSSUM scoring systems are well-established, frequently used and have been developed or validated in large patient cohorts [7, 9–14, 25–28]. The MELD Score showed heterogenic predictive power in varying cohorts of patients undergoing hepatectomy [29, 30]. The P-POSSUM scoring systems include patient’s physiological condition and operative parameters and were found suitable for predicting postoperative mortality after hepato-biliary-pancreatic surgery [6].

The current study identifies a precise prediction of the postoperative mortality risk by the previously suggested Mortality Risk Score compared to the labMELD and P-POSSUM scoring systems. Depending on the individual risk profile an increasing risk for 90-day mortality could be identified with high predictive power. In contrast, the labMELD Score showed quite heterogenic results with medium predictive power. One has to recognize that despite increasing score points the mortality risk was evaluated to be not linear increasing as one might have suspected. The predictive power of the P-POSSUM Score was low with the OS clearly underperforming.

When the labMELD Scores, the PS and OS as well as the whole P-POSSUM system were categorized according to the suggested Mortality Risk Score groups it could be demonstrated that the median labMELD as well as the median PS/P-POSSUM Score values increase gradually within the different risk groups and were able to dynamically reflect low risk to high risk patients. However, further studies are needed; especially on eastern populations.

In summary, the trend of expanding the pool of patients undergoing liver resection towards high risk surgical patients continues. The 90-day mortality rate improved over the past decade but still varies across cohorts. Overall, risk scores will never replace comprehensive preoperative assessment and proper patient selection criteria. However, they might help to estimate the individual risk profile and support planning of a risk adapted operation strategy including pre-treatment to enhance the functional liver reserve, staged resections or combined surgical and interventional radiological concepts to reduce the postoperative morbidity and mortality rates. This will not only have significant implications on the patients’ quality of life but also on healthcare-economics.

## Conclusions

The Mortality Risk score shows a precise prediction of the risk of postoperative mortality after liver surgery and should be used at the selection stage. The well-established labMELD Score estimates the postoperative mortality risk with a medium predictive power, the standard P-POSSUM Scores fail in predicting the 90-day mortality after liver resection.

## Data Availability

The datasets used and/or analysed during the current study are available from the corresponding author on reasonable request.

## Abbreviations

ASA: American Society of Anesthesiologists
AUC: Area under the curve
BMI: Body Mass Index
CI: Confidence interval
E-PASS: Estimation of Physiologic Ability and Surgical Stress
GGT: Gamma-Glutamyltransferase
INR: International Normalized Ratio
IQR: Interquartile range
labMELD: Calculated Model for End-Stage Liver Disease
MELD: Model for End-Stage Liver Disease
ME-PASS: Modified Estimation of Physiologic Ability and Surgical Stress
OR: Odds ratio
OS: Operative severity score
P-POSSUM: Portsmouth-Physiological and Operative Severity Score for the enUmeration of Mortality and morbidity
PS: Physiological score
ROC: Receiver operating characteristic
SD: Standard deviation
